# Some Twist of Molecular Circuitry Fast Forwards Overnight Sleep Hours: A Systematic Review of Natural Short Sleepers' Genes

**DOI:** 10.7759/cureus.19045

**Published:** 2021-10-25

**Authors:** Ji Hyun Yook, Muneeba Rizwan, Noor ul ain Shahid, Noreen Naguit, Rakesh Jakkoju, Sadia Laeeq, Tiba Reghefaoui, Hafsa Zahoor, Lubna Mohammed

**Affiliations:** 1 Department of Research, California Institute of Behavioral Neurosciences & Psychology, Fairfield, USA; 2 Medicine, Shanghai Medical College of Fudan University, Shanghai, CHN

**Keywords:** short sleep, adrb1, mglur1, npsr1, dec2, natural short sleeper, familial natural short sleeper

## Abstract

This systematic review focuses on different genetic mutations identified in studies on natural short sleepers, who would not be ill-defined as one type of sleep-related disorder. The reviewed literature is from databases such as PubMed*, *PMC, Scopus, and ResearchGate. Due to the rare prevalence, the number of studies conducted on natural short sleepers is limited. Hence, searching the search of databases was done without any date restriction and included animal studies, since mouse and fly models share similarities with human sleep behaviors. Of the 12 articles analyzed, four conducted two types of studies, animal and human (cross-sectional or randomized-controlled studies), to testify the effects of human mutant genes in familial natural short sleepers via transgenic mouse or fly models. The remaining eight articles mainly focused on one type of study each: animal study (four articles), cross-sectional study (two articles), review (one article), and case report (one article). Hence, those articles brought different perspectives on the natural short sleep phenomenon by identifying intrinsic factors like *DEC2*, *NPSR1*, *mGluR1*, and *β1-AR* mutant genes. Natural short sleep traits in either point-mutations or single null mutations in those genes have been examined and confirmed its intrinsic nature in affected individuals without any related health concerns. Finally, this review added a potential limitation in these studies, mainly highlighting intrinsic causes since one case study reported an extrinsically triggered short sleep behavior in an older man without any family history. The overall result of the review study suggests that the molecular mechanisms tuned by identified sleep genes can give some potential points of therapeutic intervention in future studies.

## Introduction and background

"Although sleep is an essential process for life, the brain circuits regulating sleep and the cellular and/or molecular mechanisms involved in this complex process are still enigmatic. Sleep or a 'sleep-like' behavior is present in virtually every animal species where it has been studied. Total sleep deprivation can be fatal, and partial deprivation of sleep has serious consequences on cognition, mood, and health." [1]

One of the most frequently asked sleep questions would be the optimal sleep hours for most individuals or the minimum sleep requirement to thrive in their lives. Although the report from Sleep Foundation [www.sleepfoundation.org] says that the average United States (US) adult sleeps about 7.4 hours per day during their work-offs [1], it also states that the optimal sleep duration for most adults is eight hours each day to sustain their health in general [2]. Prolonged periods of short sleep hours either due to social needs or comorbid pathologic conditions will gradually deteriorate the quality of life as a whole. Most individuals will suffer from daytime sleepiness and ultimately need to catch up with their missed sleep hours during work-offs [3].

In many pieces of literature, the term "short sleep" is often referred to synonymously as sleep deprivation or sleep-wake disorder leading to other chronic medical consequences. However, it is a less popular concept that this term can be composed of two different phenomena of sleep: short sleep phenotype and deprived sleep. The former group is also classified as natural short sleepers (NSS) who usually sleep four to six hours a day without any negative impact on health or daytime drowsiness [2]; meanwhile, insufficient sleep is a pathologic condition needed to be managed and corrected. NSS is also not to be confused with the term familial advanced sleep phase (FASP), or advanced sleep phase (ASP), in which affected individual displays spontaneous early sleep onset and offset times while overall sleep duration is unaffected [4]. Hence, as the topic of sleep is more extensively studied by researchers, the need for clarification of the lexicon of sleep is rising, and it must be handled carefully [5]. 

Other challenges of studying NSS include (i) screening of other sleep-wake variants with short sleep patterns related to the subject's underlying comorbid medical condition or pharmacological influence [6], (ii) reliability of subjective reports of short sleep patterns, which need to be confirmed by objective measurements conducted by wrist actimetry sensors and home EEG units to detect sleep/wake transition and quality of sleep [1], and (iii) recruitment difficulty due to undetected NSS cases due to subject's perception of own phenotypic short sleep behavior as non-problematic and dependence on research subject's interest in sleep research and active involvement [2]. While facing such limitations in human studies, many NSS phenotype research is based on animal studies compared to human sleep behaviors and gene sequencing. The molecular mechanisms of identified genes regulating circadian rhythm, neuronal excitability, neurotransmission, and metabolism can make a good parallel to those of humans with a well-conserved molecular basis of sleep throughout evolution [7,8].

With recent growing interest in developing medical interventions to sleep disturbances and sleep deprivation to alleviate sleep debts and associated pathologies, it is crucial to understand gene-based modulations of sleep quality and quantity, and thus overall sleep homeostasis [1]. It is only after obtaining such solid understanding that outlines for future therapeutic intervention can be drawn. This systematic review, therefore, can give a good overview of the underlying molecular mechanisms of sleep dynamics in NSS ruled by currently identified mutant alleles of *DEC2* (basic helix-loop-helix proteins differentiated embryo chondrocyte 2), *NPSR1 *(neuropeptide S receptor 1), *mGluR 1* (metabotropic glutamate receptors of subtype ), and *β1-AR* (β1-adrenergic receptor) genes in existing reports. 

Method

**Figure 1:**
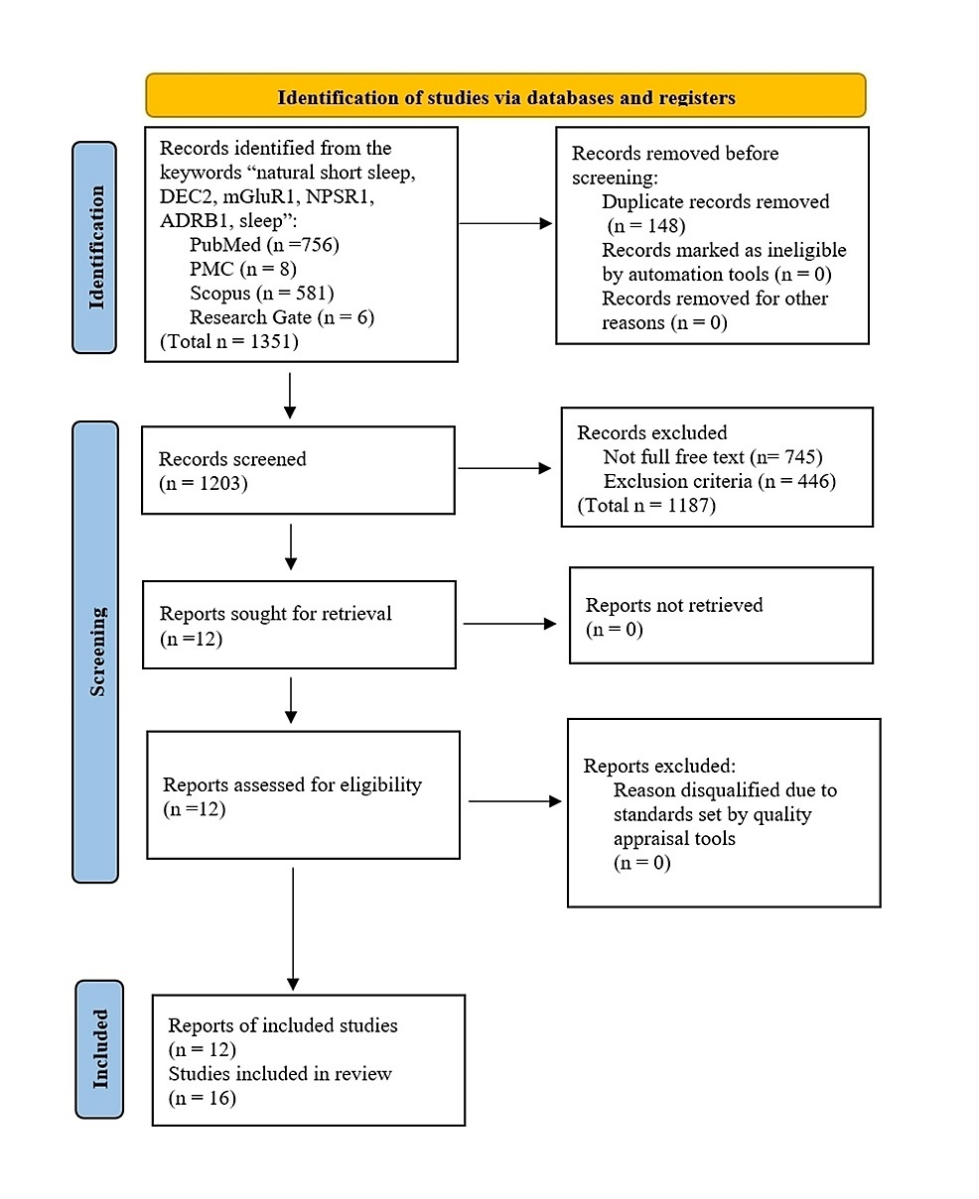
Preferred Reporting Items for Systematic Reviews and Meta-Analyses (PRISMA) Guideline Flow Diagram 2020 Adapted from: Page MJ et al. [9] DEC2: transcription factor basic helix-loop-helix proteins differentiated embryo chondrocyte 2; NPSR1: neuropeptide S receptor 1; mGluR 5: metabotropic glutamate receptors of subtype 5; ADRB1: β1-adrenergic receptor; PMC: PubMed Central; n: number of studies.

For this systematic review, keywords such as sleep, short sleep phenotype, natural short sleep, DEC2, NPSR1, mGluR1, or ADRB1 were used to search in databases including PubMed, Scopus, PubMed Central (PMC), and ResearchGate. MeSH (Medical subject headings) keyword strategies in PubMed were built for the precise result outcome: each keyword describing gene types such as "DEC2", "NPSR1", "mGluR1", and "ADRB1", had been paired with the keyword "sleep," linked by Boolean AND so that the results would be restricted to each gene's function on sleep, out of many other known functions. In addition, the keyword "natural short sleep" was used for each database to detect any potentially missed articles. We searched the relevant articles offered for free with full text extensively without limits on publication dates or study types: articles reviewed include animal studies, cross-sectional studies, a review paper, and even a case report. Our inclusion criteria were set as follows: (i) discussion on natural short sleep phenotype or familial short sleep at least some extent, if not all, or (ii) studies on the regulation of sleep duration or sleep homeostasis on a molecular genetic basis. We removed any duplicates of searched articles from different databases by importing the article list to the Endnote and using its "find duplicate" tool. We strictly excluded those articles on sleep disorders like sleep deprivation, shift work sleep disorder, or advanced sleep phase syndromes, for the precision of our analysis on the term "short sleep phenotype," which is not considered a disease. While some articles were added and cited from the articles of our direct search by using their citation index to supplement the discussion section of our review, no further searching methods like contacting authors, experts, or manufacturers, were sought. Every step taken during this screening process of searched articles followed the PRISMA guideline 2020 as Figure 1 [9] below and the same search method was repeated weekly to stay updated with its last updated search done on September 7, 2021. Lastly, we went through the PRISMA-S checklist 2021 to ensure the good quality of review content. 

Results

Initial search results from all databases mentioned were overall 1351 articles, of which 148 duplicates were found. Out of the remaining 1203 articles after duplicate elimination, only 458 records were offered in free full text and chosen to be further screened. Application of our inclusion and exclusion criteria yielded the final 16 articles. Two independent reviewers then assessed these articles via different quality appraisal tools depending on study types of each article: Joanna Briggs Institute (JBI) tools for analytical cross-sectional study (five articles), case report (one article), and randomized-controlled study (one article); the Scale for the Assessment of Narrative Review Articles (SANRA) for narrative reviews (one article); and the Systematic Review Centre for Laboratory Animal Experimentation (SYRCLE) risk of bias tool for animal study (eight articles). Among those, three articles conducted both cross-sectional studies and animal studies, and one article included both randomized-controlled trials and animal studies. Hence, they were evaluated twice for each type of study they discussed by corresponding quality assessment tools. Because no concrete rules on rating the risk of bias are given to all the tools being used, the reviewers' judgment on ratings for scores earned by studies had been made accordingly on each tool as indicated in Table 1, Table 2, Table 3, Table 4, and Table 5. As a result, we could confirm that all the articles assessed through JBI, SANRA, and SYRCLE's tools were of low risk of bias, except three animal studies that were of moderate risk of bias according to SYRCLE's risk of bias tool [10-12]. The final decision was made to include all 12 articles (with a total of 16 studies) for analyses in our systematic review. A brief overview of key findings from each study included in this review is outlined in Table 6.

**Table 1:** Joanna Briggs Institute (JBI) Checklist for Cross Sectional Studies Adapted from: Critical Apprisal Tools [10] Study reached equal or more than 70% of yes rated as low risk bias; between 69-50% of yes as moderate risk; and up to 49% or below as high risk.

Study		ITEM 1	ITEM 2	ITEM 3	ITEM 4	ITEM 5	ITEM 6	ITEM 7	ITEM 8	% Yes	Average Risk*
He et al. [1]	2009	Yes	Yes	Yes	Yes	Yes	Unclear	Yes	Yes	87.5	Low
		Yes	Yes	Yes	Yes	Yes	Unclear	Yes	Yes	87.5	
Pellegrino et al. [13]	2014	Yes	Yes	Yes	Yes	Unclear	Unclear	Yes	Yes	75	Low
		Yes	Yes	Yes	Yes	Yes	Unclear	Yes	Yes	87.5	
Spada et al. [14]	2014	Yes	Yes	Yes	Yes	Yes	Yes	Yes	Yes	100	Low
		Yes	Yes	Yes	Yes	Yes	Yes	Yes	Yes	100	
Xing et al. [15]	2019	Yes	Yes	Yes	Yes	Unclear	Yes	Yes	Yes	87.5	Low
		Yes	Yes	Yes	Yes	Yes	Yes	Yes	Yes	100	
Shi et al. [2]	2021	Yes	Yes	Yes	Yes	Unclear	Unclear	Yes	Yes	75	Low
		Yes	Yes	Yes	Yes	No	Unclear	Yes	Yes	75	

**Table 2:** Joanna Briggs Institute (JBI) Checklist for Case Reports Adapted from: Critical Apprisal Tools [10] Study reached equal or more than 70% of yes rated as low risk bias; between 69-50% of yes as moderate risk; and up to 49% or below as high risk.

Study		ITEM 1	ITEM 2	ITEM 3	ITEM 4	ITEM 5	ITEM 6	ITEM 7	ITEM 8	% Yes	Average Risk*
Seystahl et al. [16]	2014	Unclear	Yes	Yes	Yes	Yes	Yes	Unclear	Yes	75	Low
		Unclear	Yes	Yes	Yes	Yes	Yes	Yes	Yes	87.5	

**Table 3:** Joanna Briggs Institute (JBI) Checklist for Randomized Controlled Trial Adapted from: Critical Apprisal Tools [10] Study reached equal or more than 70% of yes rated as low risk bias; between 69-50% of yes as moderate risk; and up to 49% or below as high risk.

Study		ITEM 1	ITEM 2	ITEM 3	ITEM 4	ITEM 5	ITEM 6	ITEM 7	ITEM 8	ITEM 9	ITEM 10	ITEM 11	ITEM 12	ITEM 13	% Yes	Average Risk*
Holst et al. [17]	2017	Yes	Unclear	Yes	No	No	Unclear	Yes	Yes	Yes	Yes	Yes	Yes	Yes	69.2	Low
		Yes	Unclear	Yes	Unclear	Unclear	Yes	Yes	Yes	Yes	Yes	Yes	Yes	Yes	76.9	

**Table 4:** Scale for the Assessment of Narrative Review Articles (SANRA) Adapted from: Baethge et al. [11] Sum score of 7 or higher  is considered as low-risk bias; 6-4 as moderate risk; and 3-0 as high risk

Study		ITEM 1	ITEM 2	ITEM 3	ITEM 4	ITEM 5	ITEM 6	Sum score	Average Risk*
Kushikata et al. [18]	2021	2	2	1	2	2	0	9	Low
		2	2	1	1	2	0	8	

**Table 5:** The Systematic Review Centre for Laboratory Animal Experimentation (SYRCLE) Risk of Bias (ROB) Tool for Animal Studies Adapted from: Hooijmans et al. [12] Sum scores in between 20-14 is defined as low risk bias; 13-7 is moderate risk; and 6-0 is high risk where "yes" scores 2, "unclear" scores 1, and "no" scores 0.

Study		ITEM 1	ITEM 2	ITEM3	ITEM4	ITEM 5	ITEM 6	ITEM 7	ITEM 8	ITEM 9	ITEM 10	Sum Score	Average Risk*
He et al. [1]	2009	No	Unclear	Unclear	Unclear	Yes	Yes	Yes	Yes	Unclear	Unclear	13	Moderate
		No	Unclear	Unclear	Unclear	Yes	Yes	Yes	No	Unclear	Unclear	11	
Shahmoradi et al. [19]	2015	Unclear	Yes	Yes	Unclear	Yes	Yes	Yes	Yes	Yes	Unclear	17	Low
		Unclear	Yes	Yes	Unclear	Yes	Yes	Yes	No	Yes	Unclear	15	
Holst et al. [17]	2017	Yes	Yes	Unclear	Unclear	Unclear	Yes	Yes	Yes	Yes	Unclear	16	Low
		Yes	Yes	Unclear	Unclear	Yes	Yes	Yes	Yes	Yes	Unclear	17	
Hirano et al. [20]	2018	Yes	Yes	Unclear	Unclear	No	No	No	Yes	Yes	Unclear	11	Moderate
		Yes	Yes	Unclear	Unclear	No	No	No	Unclear	Yes	Unclear	10	
Shi et al. [21]	2019	Yes	Yes	Unclear	Unclear	Unclear	Unclear	Yes	Yes	Yes	Unclear	15	Low
		Yes	Yes	Yes	Yes	Yes	Yes	Yes	Yes	Yes	Unclear	19	
Xing et al. [15]	2019	Yes	Yes	Yes	Yes	Yes	Yes	Yes	Yes	Yes	Unclear	19	Low
		Yes	Yes	Yes	Yes	Yes	Yes	Yes	Yes	Unclear	Unclear	18	
Aguilar et al. [22]	2020	Unclear	Yes	Unclear	Unclear	Unclear	Unclear	Unclear	Yes	Yes	Unclear	13	Moderate
		Unclear	Yes	Unclear	Unclear	Unclear	Unclear	Unclear	Yes	Yes	Unclear	13	
Shi et al. [2]	2021	Yes	Yes	Unclear	Unclear	Yes	Unclear	Yes	Yes	Yes	Unclear	16	Low
		Yes	Yes	Unclear	Unclear	Yes	Unclear	Yes	Yes	Yes	Unclear	16	

**Table 6:** Findings of the Studies Included in the Analysis hDEC2 (also known as BHLHE41): basic helix-loop-helix family member e41 in human; CLOCK: circadian locomotor output cycles kaput: BMAL1: Brain and Muscle ARNT-Like 1; SNP: single nucleotide polymorphism; EEG: electroencephalography; DEC1: transcription factor basic helix-loop-helix proteins differentiated embryo chondrocyte 1 (also known as SHARP2/BHLHE40); DEC2: transcription factor basic helix-loop-helix proteins differentiated embryo chondrocyte 2 (also known as SHARP1/BHLHE41); WT: wild-type; IGF2: insulin-like growth factor 2; NPSR 1: neuropeptide S receptor 1; NPS: neuropeptide S; FNSS: familial natural short sleep; KO: knock-out; Npsr1: mouse NPSR1 gene; mGluR1: metabotropic glutamate receptor subtype 1; GRM1: metabotropic glutamate receptor 1 gene; Grm1: mouse GRM1 gene; Tango assay: standard arrestin-recruitment assay; mGluR5: metabotropic glutamate receptor subtype 5, HT: heterozygous; PET: positron emission tomography; MRS: magnetic resonance spectroscopy; EMG: electromyogram; PCR: polymerase chain reaction; NREM: non-rapid eye movement; REM: rapid eye movement; ADRB1: β1-adrenergic receptor gene; Adrb1: β1-adrenergic receptor gene in mice; N/A: Not Available.

Author(s) (Yr of Pub.)	Design of Study	Study Purpose	Sample Size	Method	Significance
He et al. (2009) [1]	Cross-sectional study & animal study	To identify genetic mutations in human short sleep phenotype carrier and construct transgenic animal models to testify the effect of this mutation on sleep duration and homeostasis.	Short sleep family (n = 7); DEC2-P385R mice (n = 5); WT mice (n = 8)	Gene sequencing of DNAs and self-reported sleep diaries from a short sleep family. Mouse and fly models.	Identified hDEC2 P385R point mutation from DNA gene sequences in two carriers of a short sleep family. DEC2 P385R transgenic mouse and fly models displayed the same short sleep patterns
Seystahl et al. (2014) [16]	Case report	To report unexpected arising of short sleep phenotype in patient who underwent ventriculostomy due to his chronic hydrocephalus.	59-year-old male	Self report, actigraphy, MRI imaging	Decompression of the patient's chronic hydrocephalus may have lowered supratentorial brain pressure in general so that adjacent structures such as the thalamus and hypothalamus might be responsible for the change in sleep need and homeostasis.
Pellegrino et al. (2014) [13]	Cross-sectional study	To identify other variants of the DEC2 gene that may induce short sleep phenotype in humans.	Acute sleep deprivation (n = 200), chronic partial sleep deprivation (n = 217)	DEC2 gene sequencing, EEG and delta power analysis, and cell-based luciferase assays.	Several mutations of the DEC2 gene were identified: p.Tyr362His, p.Pro384Arg, p.Pro384Gln, and p.Ala380Ser. Those mutations reduce the total sleep of carriers, and clock mechanisms involving DEC2 and CLOCK/BMAL1 transactivation must play a role in this alteration.
Spada et al. (2014) [14]	Cross-sectional study	Some objective measurements are used to examine the influence of SNP rs324981 in NPSR1 on sleep regulation.	Caucasian subjects aged between 62-79 yrs (n = 393)	Actigraphy assessment and genotyping by the TaqMan OpenArray System.	Compared to A-allele carriers, T-allele carriers reported no change in rest and sleep onsets but had much shorter rest and sleep durations. These findings paralleled with previous animal studies in mice and human reports.
Shahmoradi et al. (2015) [19]	Animal study	To investigate the relationship between DEC1/DEC2 transcription factors and cognitive function as sleep is known to closely affect memory consolidation.	WT mice (n = 4~30); DEC1/DEC2 double null mutant mice (n = 4~30)	Mouse models. Plasmid construct of IGF2 expression, brain slice analysis (Western blot analysis and immunostaining), enzyme immunoassay, antibodies, RNA extract analysis, and behavioral analysis.	DEC1 and DEC2 single null mutants did not display any cognitive dysfunctions and thus confirmed functional redundancy of both proteins. Double null DEC1/DEC2 mutants showed enhanced remote fear memory consolidation; however, deterioration of fear memory formation in aging double null mutants implied that chronic elevation of IGF2 signaling might damage cognitive processing.
Holst et al. (2017) [17]	Randomized-control trial & animal study	To identify the contribution of mGluR5 to sleep-wake homeostasis.	Healthy young men (Total n =26, control n = 9, sleep deprived n = 17); WT-mice (n = 6~10); HT-mice (n = 6~10); KO-mice (n = 6~10)	Polysomnographic and EEG recordings, PET imaging, MRS data. Mouse models (EEG/EMG signals, vigilance analysis, spectral analysis, PCR probing of brain total RNA, and Y-maze behavior analysis).	Upon sleep deprivation, there had been increased bioavailability of mGluR5 in human subjects with the presence of objective markers of sleep need (delta and low frequency <1Hz EEG activity), and mGluR5 KO mice showed severely disturbed sleep homeostasis with poor adaptation to new tasks, and lack of recovery sleep.
Hirano et al. (2018) [20]	Animal study	To confirm that mutant DEC2 gene (P384R) shortens sleep duration by modulating neuropeptide hormone orexin.	hDEC2-WT mice (n = 4~8); hDEC2-P384R mice (n = 4~8)	Mouse models (backcrossed to C57BL/6J >10 generations), Cell-based luciferase assay, and immunoprecipitation using plasmid vectors.	Mutant DEC2 showed decreased transcriptional repressor activities for orexin expression, thus possibly increasing total arousal states in mammals. DEC2 must be responsible for sleep-duration regulation, at least to some extent, by fluctuating orexin levels.
Shi et al. (2019) [21]	Animal study	To identify additional genes affecting sleep duration to broaden the knowledge of gene and molecular pathways essential for sleep homeostasis in humans.	FNSS family with ADRB1-A187V carriers (n = 15); WT-mice (n = 4~20); Adrb1-mutant mice (n = 4~19)	Self-reported sleep diary, SNP genotyping, and exome sequencing. Mouse models (protein extraction and immunoblot analysis, RNA extract PCR analysis, stereotaxic surgery, EEG recording, maze-monitoring, etc.).	Mutant ADRB1 was identified in humans with FNSS trait. Mutant Adrb1-A187V mice showed reduced sleep time of 55 minutes per day and increased active time, while human carriers slept 2 hours per day less on average than noncarriers. Hence, ADRB1 mutant neurons in dorsal pons may have increased wake-promoting activities leading to short sleep traits.
Xing et al. (2019) [15]	Cross-sectional study & animal study	To identify mutant NPSR1 gene in the FNSS family and recreate it in mouse models to confirm the role of the NPSR1/NPS system in sleep regulation.	Carriers in a FNSS family (n=2); Npsr1-KO mice (n = 3); Npsr1-Y206H mice (n = 7~12); WT Npsr1 mice (n= 8~19)	Exome sequencing of FNSS family and mouse models (backcrossed to C57BL/6J minimum four generations, maze-monitoring, EEG analysis, and mice brain slices and analysis).	As a gain-of-function mutation inducing arousal, NPSR1-Y206H transgenic mice had reduced sleep duration similar to human FNSS human subjects. This finding further strengthens the importance of the NPS/PNSR1 system in control of sleep duration.
Aguilar et al. (2020) [22]	Animal study	To examine the functions of mGluR5 involved in neural oscillations during sleep, which may reproduce schizophrenia-like behaviors in the absence.	WT mice (n = 8~9); mGluR5-KO mice (n = 6~12)	Mouse models: stereotaxic surgery, EEG analysis (NREM and REM sleep), and a visual analog to the acoustic steady-state response (ASSR) for task-evoked gamma power.	mGluR5 KO mice showed loss of sleep spindle density, wake alpha power, and 40-Hz visual task-evoked gamma power and phase locking. Also, a reduction in sigma power (10-15Hz) was observed during NREM sleep transitions. Overall, mGluR5 KO mice reproduced those EEG signals much like to schizophrenia patients.
Kushikata et al. (2021) [18]	Review	To investigate the role of NPS in anesthesia, analgesia, and sleep-wake modulation to pursue better medical care.	N/A	N/A	NPS and orexin promote emergence from anesthesia but do not necessarily inhibit the sedation by anesthesia. Hence, anesthetic induction and emergence may not share the same mechanism of action. Also, NPS attenuates not only inflammatory pain via noradrenergic neuronal activity and dopaminergic neurons but also other kinds of pain like neuropathic pain via micro-infusion of NPS into the amygdala. As an indirect inhibitor of sleep-inducing neurons, NPS acts as a wake-promoting substance by activating histaminergic and orexinergic neurons.
Shi et al. (2021) [2]	Cross-sectional study and animal study	To study the function of mGluR1 in sleep-wake regulation by examining mutant GRM1 carriers and mouse models.	GRM1-K50230 family (n = 9); GRM1-K07331 family (n = 4); WT-mice (n = 16~19); Grm1b R889W mice (n = 4~11); Grm1 S458A mice (n = 4~23)	Exome sequencing and Mouse models (protein extraction and immunoblot analysis, Tango assay, EEG analysis, electrophysiological recording, and RNA sequencing).	Two loss-of-function mutations of GRM1 with the short sleep phenotype had been identified in two FNSS families: K50230 and K07331. Mutant mice displayed a reduction in sleep, and this confirmed role of mGluR1 in sleep regulation.

## Review

Continuous studies of NSS from various angles have found a familial tendency of such a trait, and that multiple genes must play a role in quantifying sleep requirements in mammals. In this review, some of the well-defined phenotypic induction of NSS trait in either animal models or human studies ruled by *DEC2*, *NPSR1*, *mGluR1*, and *β1-AR *gene variants are discussed in separate sections as below.

Induction of short sleep trait by different single point-mutant variants of *DEC2*

**Figure 2:**
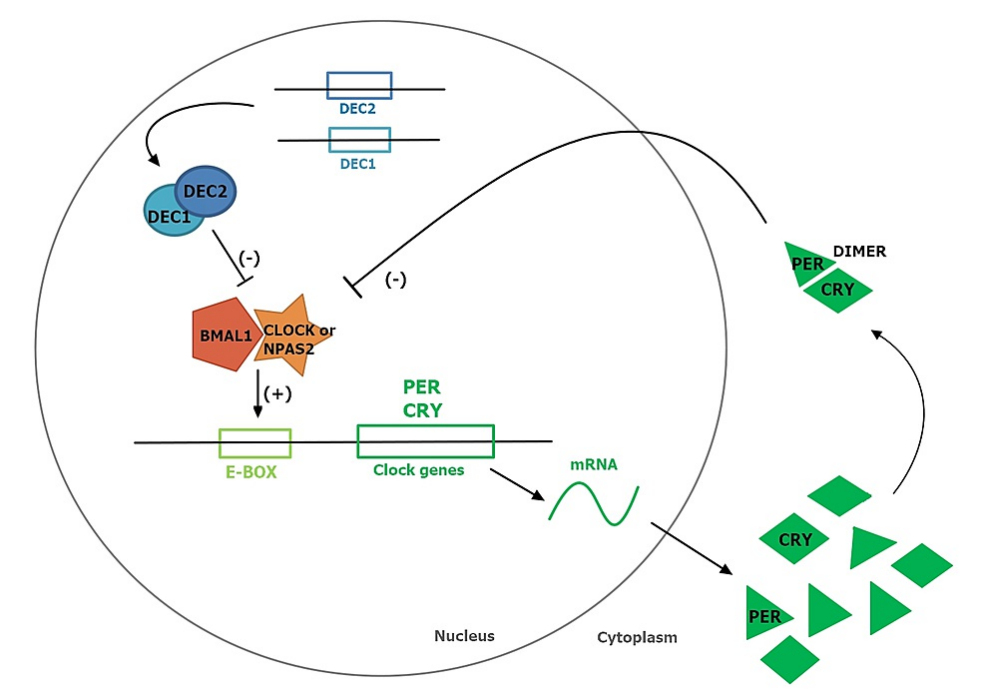
Abundance of PER and CRY Proteins Fluctuates According to Circadian Rhythms DEC 1:  transcription factor basic helix-loop-helix proteins Differentiated Embryo Chondrocyte 1; DEC2: transcription factor basic helix-loop-helix proteins Differentiated Embryo Chondrocyte 2; BMAL1: transcriptional activator Brain and Muscle Arnt-Like protein-1; CLOCK, transcriptional activator Circadian Locomotor Output Cycles Kaput; NPAS2: transcription factor Neuronal PAS domain protein 2; PER, repressor Period protein; CRY, repressor Cryptochrome protein.

Among many other circadian modulating genes like Period Circadian Regulator 2 (*PER2*), neuronal PAS domain protein 2 (*NPAS2*), aryl hydrocarbon receptor nuclear translocator-like/ Brain and Muscle ARNT-Like 1 (*ARNTL/BMAL1*), circadian locomotor output cycles kaput (*CLOCK*), and Cryptochrome Circadian Regulator 1 and 2 (*CRY1/CRY2*), several studies have found that *DEC2* gene is closely related to induction of short sleep duration [1]. CLOCK and BMAL1 transcription factors bind to each other as a heterodimer to activate enhancer box (*E-box*) element in promotor regions of period genes (*PER* genes). DEC2, as part of the same transcription family, interacts and suppresses CLOCK and BMAL1 via direct protein-protein interaction with BMAL1 and/or competition for *E-box* element as briefly described in Figure 2 [23].

An hDEC2 point mutation (P385R) in two affected individuals in a small family first had been identified by He et al. [1]. They displayed a distinct difference in sleep duration from the norm: their habitual shorter sleep time reported was an average of 6.25 hours compared to an average of 8.06 hours in noncarrier family members. Therefore, it was speculated that the specific mutations in the *DEC2* gene, which suppress the *CLOCK/BMAL1 *activation, induce phenotypical short sleep [13]. Further study on the point mutation in *DEC2* gene was done in both mouse and fly models by comparing wild-type (WT) and mutant carrier (DEC2*-*P385R) of missense mutation replacing a proline by arginine in amino acid position 385 in exon 5 of *DEC2* [1]. Compared to the WT group, the DEC2-P385R group demonstrated more wakefulness with both shorter non-rapid eye movement (NREM) and rapid eye movement (REM) during sleep and thus shorter sleep duration. Also, sleep deprivation in the DEC2-P385R mice led to slow and incomplete NREM sleep recovery with only slight NREM delta power enhancement implying adaptation to shorter sleep with altered sleep homeostasis [1]. 

In the study conducted by Pellegrino et al. with luciferase assay and plasmid construction, more variants of the *DEC2* gene, yielding p.Tyr362His, p.Pro384Arg, p.Pro384Gln, and p.Ala380Ser were discovered and compared with WT *DEC2* gene [13]. In addition to a single variant, p.Pro384Arg had been identified previously by He et al. [1], and a variant p.Ala380Ser had been rarely detected in the European from Centre d'Etude du Polymorphisme Humain (CEPH) population without any known sleep data, p.Tyr362His, and p.Pro384Gln were two newly found variant types in their study [13]. Among four DEC2 variants examined, only p.Pro384Gln did not significantly affect its molecular activities compared to WT. However, variants type p.Tyr362His, p.Pro384Arg and p.Ala380Ser showed altered repression activity of DEC2 on CLOCK/BMAL1 activation and would likely shorten the sleep duration and alleviate impacts of sleep deprivation [13]. Furthermore, those variants in DEC2 also had the same effect on the NPAS2-BMAL1 complex [13] where NPAS2, another binding partner of BMAL1, is known to alter sleep-wake quantities and act upon stimuli other than light, in some restricted feeding, as found in studies of NPAS2 knock-out mice [24,25]. Therefore, this finding left an open-ended question of whether DEC2 variants show their functional effects through interaction with CLOCK or NPAS2.

As He at el. [1] previously introduced a family with natural short sleep trait in their study, similar findings were illustrated in twin study by Pellegrino et al. [13]. The carrier twin had a much shorter total sleep time (299.3 min) than his noncarrier brother (364.7 min); meanwhile, no difference in NREM sleep duration was noted. Along with higher delta power, a measure of sleep drive, the carrier twin also showed minimal behavioral and metabolic impairment in response to sleep loss compared to the noncarrier by reporting significantly fewer average lapses of performance alertness and almost the same BMI reading (23.9 kg/m2) as his brother (25.3 kg/m2) [13]. This phenomenon could be due to quicker neuro-hormonal changes resulting in a sharp termination of increasing nocturnal melatonin levels and then an earlier rise of morning cortisol levels in the short sleeper in a sleep-deprived setting as compared to long sleepers [26], despite ambiguity in underlying molecular mechanisms. 

It had been reported that double null mutation of *DEC1/DEC2* brings complete loss of DEC function in mice. This complete loss-of-function alters sleep architecture with substantially attenuated light-to-dark amplitude of the different levels of alertness and decline of working memory, unlike single mutant DEC2-P385R mice displaying short sleep phenotype without any cognitive dysfunctions [1]. A study of DEC1 and DEC2 mutations further dove into its relationship with sleep homeostasis and memory consolidation [19]. The transformation of recent fear memory in the hippocampus (Hi) into long-term memory in the cortex relies on different sleep aspects like the sleep-dependent memory replay between the Hi and the cortex [27,28]. In the Hi, mitogen-activated protein kinase (MAPK) participates in the processes of long-term memory formation, while in the forebrain, NPAS2 is involved in NREM sleep regulation and Hi-dependent cognitive processing [29-31].

In a study [19], the single and double null mutant of *DEC1* and *DEC2* genes, as a part of homeostatic sleep modulators, were introduced in mouse models to observe their effects on cognitive processes. The double null mutants displayed enhanced cortex-dependent remote fear memory formation presumably due to elevated insulin-related growth factor 2 (IGF2) expression leading to MAPK signal activation in the anterior cingulate cortex without any change in Hi-dependent recent memory formation [19]. This finding implied that underlying mechanisms of working memory (executive functions of the brain) and long-term memory formation must be distinct and altered differently in double *DEC1/DEC2* mutant mice [19,32]. Moreover, as the mutant mice age, they showed a significant decline in memory performance which may be explained, though somewhat unclearly, by lack of DEC1/DEC2 functions or chronic increase of IGF2 expression levels [19]. Lack of such cognitive shortage observed in the double mutant, single mutant DEC2-P385R protein might not simply act in a dominant-negative manner interrupting DEC1/DEC2 repressor functions by heterodimerization with WT protein [33,34] and have its specific repressive functions by interacting with CLOCK/NPAS2 along with other transcriptional regulators [13]. 

Orexin, also called hypocretin (Hcrt), is a neuropeptide known for its role in regulating arousal [35]. Lower circulating Hcrt and fewer Hcrt receptors cause decreased arousal at certain times of day and fragmentation of sleep architecture [35]. In a further study of the role of DEC2 protein [20], DEC1 and DEC 2 also form a complex with MyoD1 and E12/47, the other set of transcription factors, to bind to another E-box sequence (CAG CTG) in the promoter region of Hcrt. In this region, DEC2 represses MyoD1 activating prepro-orexin gene expression. Therefore, fluctuations of Hcrt levels are controlled by DEC2 expressions oscillating in a circadian rhythm in the hypothalamus and cerebrospinal fluid [35,36]. In vitro studies [35] found that altered binding of mutant DEC2-P384R to E12 and DNA resulted in the reduced repressive activity of DEC2, and it was suggested that MyoD1/E12 heteromer involved in the recruitment of DEC2 to the binding site, E-box. Interestingly, DEC2 effect cancellation can occur due to overexpressed E12 or E47, which might be competing with MyoD1 binding to E-boxes, and thus unable to form a heteromer (MyoD1/E12) for the DEC2 recruitment. Or it might be due to the inability of DEC2 to replace overexpressed E12/47 in E-box binding sites. Hence, this DEC2 identified function in sleep regulation via Hcrt could make DEC2 a good target for modulating orexinergic signaling and could be used in inventing new treatment of insomnia patients [20]. 

NPS/NPSR1 signaling and reduction of sleep duration by a gain-of-function mutation in NPSR1

**Figure 3:**
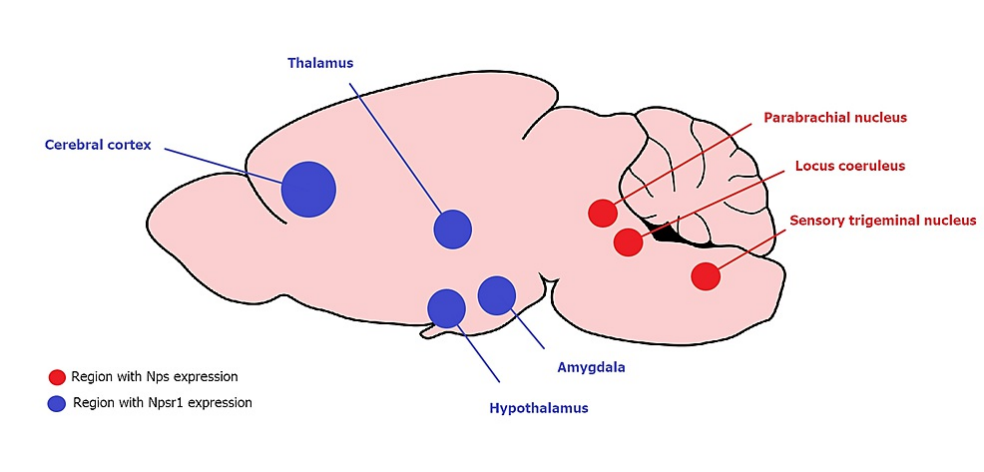
Some Major Regions Where Nps and Npsr1 Are Highly Expressed in Mouse Brain (From the Sagittal View) Nps: neuropeptide S mouse gene; Npsr1: neuropeptide S receptor 1 mouse gene.

The neuropeptide S (NPS) is highly expressed in brainstem neurons adjacent to the locus coeruleus in the parabrachial nucleus and principal sensory trigeminal nucleus [14,37,38]. The ascending arousal network is distributed over the locus coeruleus and the parabrachial nucleus areas, while the trigeminal sensory nucleus is also influenced by the sleep-wake cycle [39,40]. Unlike specific distributions of NPS, neuropeptide S receptor (NPSR1), a G-protein coupled receptor, is more extensively distributed throughout the brain: it can be primarily found in the sleep-wake system of the hypothalamus and thalamus and also observed in the cortex and the amygdala [14] as illustrated in Figure 3. More importantly, NPSR1 can be found in some areas for sleep induction and in hypothalamic areas such as the perifornical region and the tuberomammillary nucleus, in which wake-promoting orexin and histamine are expressed, respectively [14,41,42]. Protein kinases are activated upon NPS binding to NPSR1, and intracellular cAMP and Ca^2+^ levels are increased in neurons expressing NPSR1 for neurotransmission modulation by NPS [37].

Most studies in the single nucleotide polymorphism (*SNP*) rs324981 (triplet position 107 of the *NPSR1* gene on chromosome 7p14.3) had been done in mouse models, making a parallel to NPS/NPSR1 functions in humans [14]. The T-allele of the *SNP* replaces the asparagine with isoleucine in the active center of the NPSR1-binding site, which consequently increases sensitivity to its ligand, NPS, up to tenfold [43]. Based on a previous similar study done by Gottlieb et al. [44] conducted via subjective measurement of sleep with two single questionnaires, Spada et al. [14] replicated the study with an objective measure of sleep via actinography. They again examined homozygous T-allele carriers who showed a reduction in sleep duration than *A-allele* carriers but had no significant difference in sleep onset. However, underlying functional mechanisms of this alteration of sleep duration were to be further investigated.

Further study on NPS/NPSR1 mutation in human and mouse models by Xing et al. [15] demonstrated the mutation NPSR1-Y206H in mice with shorter sleep duration as well as resistance to memory loss in response to sleep deprivation, and similar corresponding homologous mutation in people with familial natural short sleeper (FNSS) trait. They stated that NPSR1-Y206H mice to be a gain of function mutation due to absence of sleep phenotype in NPSR1 knock-out (KO) mice and their better response upon NPS administration inducing wakefulness and hyperactivity. NPS also has been known to facilitate learning and memory [45], elicit anxiolytic [37], and have antinociceptive effects [46]. Hence, other than the short sleep trait, NPSR1-Y206H could also be responsible for at least some of the seemingly protective traits for carrier subjects, although it is yet unknown that whether these traits are from efficient sleep or regulation by different NPS/NPSR1 signaling. These findings, as a result, make NPS/NPSR1 system a good therapeutic target for sleep improvement and treatment for sleep-related disorders in humans [15]. 

In various sites of its action, NPS administration can facilitate emergence from anesthetic agents without interrupting their sedative effects [47]; attenuate inflammatory or neuropathic pain via noradrenergic and dopaminergic neurons, or amygdala [48], respectively; and also modulate sleep-wake status through histaminergic and orexinergic neurons [18]. GABAergic neuron with its inhibitory function in sleep regulation, in the ventrolateral preoptic area (VLPO), is facilitated by NPS, which works as an indirect inhibitor of sleep-inducing neurons in this manner. In the other study, NPS administration (0.1 and 1nmol, icv) markedly raised wakefulness for about two hours of duration, and it was evaluated by increased high-frequency activities (14.5-60 Hz) detected in EEG measurement [49]. Induction of c-Fos (Fos proto-oncogene) expression by NPS injection in areas of abundant NPSR1 expressions such as histaminergic neurons and orexinergic neurons promote wakefulness and quantified sleep rhythm in sleep-wake associated brain structures [18]. Though with yet unknown mechanism, endogenous NPS showed inhibitory action on NREM sleep during light phase but no effect on REM sleep, implying that those two sleep statues must be regulated differently [50].

Loss-of-function mutations in mGluR1 and mGluR5 subtypes lead to different outcomes

**Figure 4:**
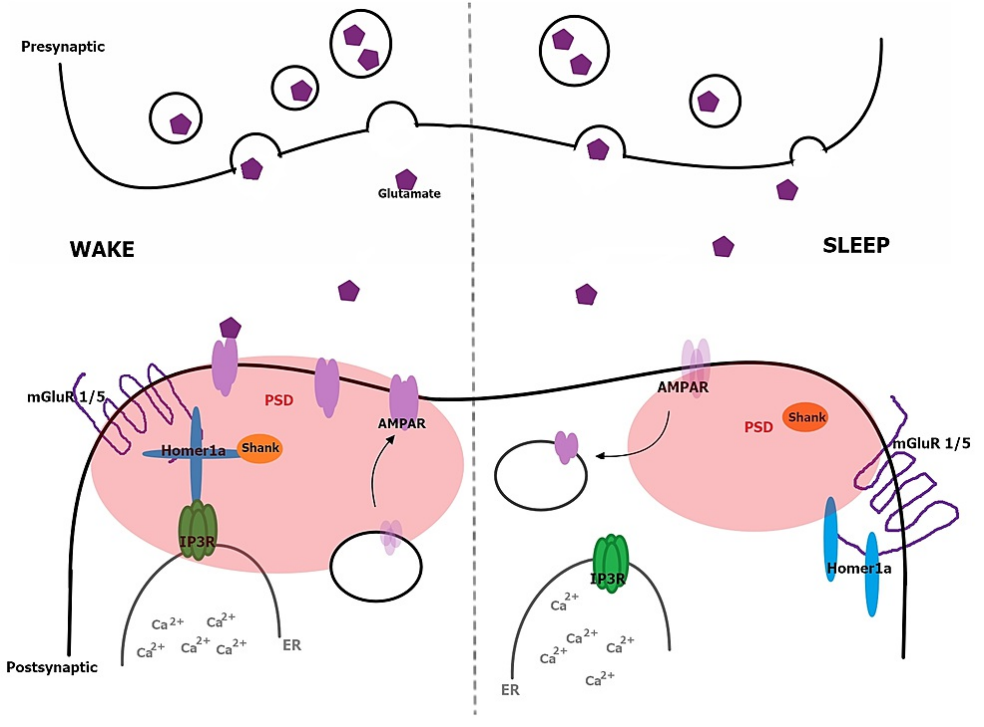
Periodic Changes of Neural Excitability and Synaptic Strength During the wake period, mGluR1/mGluR5 binds to long-form Homer proteins (tetramer of Homer1a) to anchor IP3R in ER and Shank at the base of PSD, thus increasing calcium release for neuronal excitability and upregulating AMPAR for strengthening synapses, respectively. During sleep, mGluR1/mGluR5 is agonized by Homer1a monomer and dissociated from IP3R and Shank, which, in turn, decreasing neural excitability and synaptic strength [51]. mGluR1/5: Group I metabotrpic glutamate receptors; Homer1a: Homer protein homolog 1 a; AMPAR: AMPA (a-amino-3-hydroxy-5-methyl-4-isoxazolepropionic acid)-type glutamate receptor; IP3R: 1,4,5-triphosphate receptor; PSD: postsynaptic density; Shank: Shank protein; ER: endoplasmic reticulum.

The group l metabotropic glutamate receptors (mGluR1 and mGluR5 subtypes) are involved in the regulation of normal cellular activity and synaptic plasticity, as well as sleep homeostasis (by mGluR5), and many of them are coupled with different Homer isoforms for binding and are distinctively regulated [51,52]. During sleep, synapses are loosened by Homer1a (sleep regulator) and mGluR1/mGluR5 via signaling of neuromodulators promoting sleep-wake cycles [53], as depicted in Figure 4.

In another study [2], two rare independent mutations, *K50230* and *K07331*, of the glutamate metabotropic receptor 1 gene (*GRM1*) had been identified in two unrelated FNSS families. *K50230* altered the arginine into a tryptophan at position 889 in the C-terminal intracellular domain of the isoform GRM1b-R889W, and *K07331* substituted the serine into an alanine at position 458 in the N-terminal extracellular domain yielding the isoform GRM1-S458A. Despite their different mutant domains, those two mutations of *mGluR1 *shared similar functional changes, but both were comparable to WT *mGluR1*. Loss-of-function mutation in mutant *mGluR1* showed decreased activation of extracellular-signal regulated kinase (ERK) pathway, which had been known to lengthen the sleep duration of mouse models; hence, shortened sleep duration with similar amounts in both mutant strains was observed in mice. The difference was that the mixture of WT and mGluR1b-S458A receptors, compared to mGluR1b-S458A alone, restored activity to a level consistent with a loss-of-function of mutant receptor whereas mixing WT and mGluR1b-R889W with 1:1 ratio showed decreased activity compared to WT or mGluR1b-R889W alone, implying a dominant-negative effect. Among variants of *mGluR1* like mGluR1a, mGluR1b, mGluR1d, and mGluR1E55, mGluR1a isoform with the longest C-terminal domain had a strong intrinsic activity that could overcome the effect of mutant S458A [2] and was playing a dominant role, which could restore all cerebellar defects of mGluR1-KO mice. At the same time, mGluR1b could show only partial recovery of such defects [54]. Nonetheless, human subjects with FNSS carried heterozygous mutations with an expectation of a 1:2:1 ratio of WT-WT, W-Mut, and Mut-Mut, respectively, and displayed decreased receptor activities in both mutant types in vivo.

The degree of shortened sleep length (~25 min less) induced by mGluR1, however, was less evident compared to other known mutations causing changes in sleep duration such as Adrb1-A187V (mouse ADRB1-A187V) (55 min less) and Npsr1-Y206H (mouse NPSR1-Y206H) (71 min less) in mice. A similar trend was noted in human models: 132 min less in ADRB1-A187V, 180 min less in NPSR1-Y206H, and ~100 min less in *GRM1* mutant carrier humans [2,15,21]. Furthermore, no change in delta power in mouse carrier models was observed, whereas both Adrb1-A187V and Npsr1-Y206H mice showed an increased delta power at the end of the active phase and beginning of the resting phase. This finding was somewhat unexpected because mGluRs have been known to regulate sleep homeostasis [53]. It is, however, essential to note that human carriers with FNSS traits are healthy without any consequences observed in the pathogenic *GRM1* mutant carriers caused either by gain-of-function or loss-of-function mutations [22,55], and thus, the activity of mGluR1 must be strictly regulated in vivo [2]. 

mGluR5 is a part of the molecular machinery controlling sleep-wake homeostasis where specific negative allosteric regulators of mGluR5 promote sleep in mice, while positive allosteric regulators promote alertness [56]. Increased mGluR5 expressions in the sleep-deprived individual were directly associated with increased sleep drive during brain imaging along with EEG biomarkers: delta and low frequency (<1Hz) EEG activity in NREM sleep for improvement of memory functions in humans [57]. Severe dysregulation of sleep-wake homeostasis is triggered in knock-out mouse models without functional mGluR5. Those mice exhibited poor behavioral adjustment to an assigned task and loss of recovery sleep after sleep deprivation [17]. Also, the appearance of delta power during alertness in baseline and recovery periods in mice with loss-of-function mGluR5 mutation was diminished, especially during the dark phases of baseline and recovery periods [17]; as a result, consolidation of contextual memory in mice was severely disturbed. These findings emphasize [58,59]. Moreover, in mGluR5 KO mice, there had been impairment of sleep spindle density, enhancement of sleep fragmentation and time spent in NREM sleep, and disruption of NREM sleep-state transitions, among which some phenomena observed were much like the ones in schizophrenia: decreased sleep spindle density and alpha power, and poor 40 Hz visual task-evoked gamma and phase locking [55]. Hence, it was suggestive that mGluR5 enhancing drugs might improve sleep spindle losses, task-evoked gamma deficits, and specific sleep difficulties in addition to alleviating more notable symptoms of schizophrenia [22,56].

*ADRB1* mutant allele carried by affected members of FNSS families

Noradrenergic signaling in the CNS has been well known to regulate sleep; however, only little is discovered about the function of β-adrenergic receptors (β-ARs) [60]. In the brain, β-ARs are thought to conduct the effect of norepinephrine (NE) on alert waking and REM sleep, and this is how β-blockers can cause prolonged sleep onset and difficulty staying asleep possibly by decreasing melatonin release [60].

With more than 50 FNSS families identified thus far, the genetic linkage data detected a rare mutation in a *β1-AR* gene (*ADRB1*), known to be ADRB1A187V, within all carriers of FNNS phenotype and found that there were increased expressions of ADRB1 in neurons of dorsal pons (DP) in which wakefulness is promoted [21]. Findings from mutant brain slices concluded that the inhibitory function of β1-AR is largely affected by reducing its agonist protein abundance, whereas the excitatory function is more tolerated against falling protein levels. As a result, the DP *ADRB1* neurons could stay more active while fewer neurons were inhibited by the natural ligand, and the wake-promoting activity could be kept high, leading to a short sleep phenotype [21]. In mouse models, on the other hand, an increase in active time and reduction in sleep duration of 55 minutes per 24 hours were observed, but these findings were rather to a lesser extent than human models. This might be due to nocturnal behaviors and adapted fragmented sleep of mice less affected by sleep length, consolidation, timing, and other sleep parameters than humans [21]. These findings together support the role of ADRB1-A187V as one of the causative mutations in FNSS and the effect of DP β-ARs in sleep-wake modulation. As a result, it would be pertinent to explore deeper into the underlying complex circuitry mechanisms of the sleep-wake cycle and potential therapeutic targets of β1-AR for managing sleep-associated illnesses [21].

Limitations

In this review, various genetic factors shortening sleep duration or sleep need in humans were studied together with their possible underlying mechanisms. However, there are some limitations on studies of NSS phenotype in humans. For instance, though there had been more examinable human subjects with NSS, the numbers are still somewhat small due to difficulty in finding such subjects with NSS traits, excluding those with pathological sleep shortages. Although animal models were incorporated in the study due to similarities shared by human and mouse models (or even Drosophila), sleep behaviors in those two models are not the same, as mentioned by Shi et al. [48]. The influences brought by sleep mutant genes may be different in different species, leading to some ambiguity.

Also, a recent case study by Seystahl et al. [16] opens a new possibility of extrinsic factors leading to NSS. In the study, they encountered a 59-year-old male patient suffering from chronic hydrocephalus, with rather an average sleep duration (seven to eight hours) [58]. After the third ventriculostomy to relieve his hydrocephalus, however, he reported a substantial decrease in sleep need of four to five hours without feeling fatigued or excessive sleepiness during daytime [16]. It was a fascinating case since the patient had no family history of NSS, and his neuropsychological examination confirmed no cognitive or physical consequences from the new onset of short sleep at least over 17 months (as per his last follow-up visit) [16]. Seystahl et al. [16] suspected that decompression of the wake-promoting histaminergic neurons in the tuberomammillary nucleus, due to their proximity to the third ventricle, might have induced altered gene expression involving sleep regulation and homeostasis [21].

As a result, intrinsic factors ruled by genetic variants discussed in this review do not fully outline the whole picture of the NSS phenomenon in humans. More extensive research in this area must be done, and novel approaches to the topic of NSS from different angles still await.

## Conclusions

Excluding from a category of sleep-wake disorders, the natural short sleep phenotype is not per se considered an illness, endangering one's health or well-being. People with the NSS trait do not experience the medical consequences driven by short sleep duration. This sleeping habit is not meant to be practiced or learned upon one's desire, but rather is an intrinsic nature that lasts lifelong. There already had been several identified genetic factors that bring out this short sleep phenomenon of humans and other mammals. To date, the most well-known genes triggering shortening of sleep duration or sleep need, if mutated, are *DEC2, NPSR1, mGluR1, *and* β1-AR *genes. Not necessarily knocked out its functions completely, each gene has its unique point-mutation, either upregulating or downregulating its function involving the circadian clock without causing any significant changes in the other biological systems. As a result, each molecular genetic mechanism underlying the NSS trait has therapeutic potential for managing deep-seated sleep-wake disorders or related cognitive dysfunctions. At a glance, this systematic review gives a general overview of accomplishments from the previous research works on NSS until today, with yet many unknowns. It, therefore, may suggest some directions in which future research on sleep could be heading towards.
